# TRPV1 Antagonists and Chronic Pain: Beyond Thermal Perception

**DOI:** 10.3390/ph5020114

**Published:** 2012-02-02

**Authors:** Michael R. Brandt, Chad E. Beyer, Stephen M. Stahl

**Affiliations:** 1 Department of Pharmacology and Physiology, Drexel University College of Medicine, Philadelphia, PA 19102, USA; 2 IteraMed L.L.C., Doylestown, PA 18902, USA; 3 Ariel Pharmaceuticals, Broomfield, CO 80021 USA; Email: cbeyer@arielpharma.com; 4 Neuroscience Education Institute, University of California San Diego, Carlsbad, CA 92008, USA; Email: smstahl@neiglobal.com

**Keywords:** allelic variants, chronic pain, mechanotransduction, neuropathic pain, osteoarthritis, Transient Receptor Potential Vanilloid 1 (TRPV1)

## Abstract

In the last decade, considerable evidence as accumulated to support the development of Transient Receptor Potential Vanilloid 1 (TRPV1) antagonists for the treatment of various chronic pain conditions. Whereas there is a widely accepted rationale for the development of TRPV1 antagonists for the treatment of various inflammatory pain conditions, their development for indications of chronic pain, where conditions of tactical, mechanical and spontaneous pain predominate, is less clear. Preclinical localization and expression studies provide a firm foundation for the use of molecules targeting TRPV1 for conditions of bone pain, osteoarthritis and neuropathic pain. Selective TRPV1 antagonists weakly attenuate tactile and mechanical hypersensivity and are partially effective for behavioral and electrophysiological endpoints that incorporate aspects of spontaneous pain. While initial studies with TRPV1 antagonist in normal human subjects indicate a loss of warm thermal perception, clinical studies assessing allelic variants suggests that TRPV1 may mediate other sensory modalities under certain conditions. The focus of this review is to summarize the current perspectives of TRPV1 for the treatment of conditions beyond those with a primary thermal sensitivity.

## Non-Standard Abbreviations

WHO: World Health Organization
TRPV1: Transient Receptor Potential Vanilloid 1
CFA: Complete Freund’s Adjuvant
DRG: Dorsal Root Ganglion
SNL: Spinal Nerve Ligation
PSNL: Partial Sciatic Nerve Ligation
L: Lumbar
CCI: Chronic Constriction Injury
CGRP: Calcitonin Gene-Related Peptide
MIA: Mono-Iodoacetate
siRNA: Small Interfering RNA
RNAi: RNA Interference
BCTC: N-(4-*tert*-butylphenyl)-4-(3-chloropyridin-2-yl)tetrahydropyrazine-1(2*H*)-carboxamide
CNS: Central Nervous System
i.t.: Intrathecal
HbSS: Human Sickle Hemoglobin
OA: Osteoarthritis

## 1. Introduction

Chronic neuropathic pain is the result of a lesion or disease that affects somatosensory processing. Despite consensus that this syndrome arises from injury to the peripheral and/or central nervous systems, the numerous mechanistic differences of the maladaptive responses to deal with pain represent a major obstacle in the development of novel and effective medications. Chronic pain disorders, including neuropathic pain, post-herpetic neuralgia, fibromyalgia, osteoarthritis, and bone cancer pain, have collectively emerged as a serious public health concern. In the United States alone, chronic pain syndromes affect 25% to 30% of the population and nearly 60% of people over the age of 65 [1]. Recent statistics from the World Health Organization (WHO) estimate that 5% of the global population, 70% of patients with cancer, and 95% of patients with spinal cord injuries suffer from some form of chronic pain. Furthermore, chronic pain disorders are often co-morbid with other diseases including cancer, metabolic disease, and psychiatric disorders including anxiety and depression [2]. Taken together, these (and other) pain disorders represent one of the most underestimated health-care burdens costing more than $200 billion in annual health-care expenses [3]. 

The Transient Receptor Potential Vanilloid 1 (TRPV1) channel is one of the most researched and targeted mechanisms for the development of novel analgesics for inflammatory pain owing to its distribution and function. These channels are predominately expressed in small sensory C-fibers and to a lesser extent in Aδ-fibers [4], both of which terminate in the spinal dorsal horn, where TRPV1 is localized to both pre- and post-synaptic neurons in lamina I and II, as well as to glial cells [5]. In addition to localization at the spinal level, TRPV1 has supraspinal localization and contributes to descending modulation of nociceptive stimuli [6]. TRPV1 is activated by heat (>43 °C), as well as endogenous eicosanoids and protons (pH < 5.9). With respect to pain, the expression of TRPV1 channels are upregulated in preclinical models of inflammation [7,8], as well as clinical inflammatory conditions, such as osteoarthritis and rheumatoid arthritis [9], inflammatory bowel disease [10,11], gastro-esophageal reflux disease [12,13], chronic pelvic pain [14], and chronic cough [15,16]. In addition to disease related changes in receptor expression, inflammatory mediators such as cytokines, prostaglandin, bradykinin, glutamate, serotonin, and nerve growth factor all have been shown to increase the phosphorylation state of TRPV1, thereby increasing channel activity [17,18,19]. Collectively, these data suggest that TRPV1 is a critical integrator of inflammatory pain signaling [20,21,22,23].

Initial approaches targeting TRPV1 utilized agonists for the treatment of pain. Capsaicin is the prototypical TRPV1 agonist that is found in many topical formulations [24]. Agonist treatment for pain is related to the initial excitation of sensory neurons followed by a refractory state of desensitization, where the neuron becomes unresponsive to TRPV1 agonists and other inflammatory mediators. Repeated or high dose application produces a reversible ablation of the nerve fiber that further reduces sensitivity to cutaneous stimuli such as tactile, heat, mechanical and cold [25]. However, because this later approach essentially produces a neurolytic lesion of TRPV1-associated epidermal nerve fibers, the physiology for pain alleviation is mechanistically different from antagonist approaches.

The discovery of selective TRPV1 antagonists has provided additional support for the role of TRPV1 channels in inflammatory pain conditions. Pre-clinically, TRPV1 antagonists are effective at blocking thermal hypersensivity to numerous inflammogens (e.g., carrageenan and complete Freund’s adjuvant; CFA), without modulating associated inflammatory responses, as measured by edema. Initial clinical results indicate that TRPV1 antagonist decrease thermal pain perception in normal subjects and elevate core body temperature [26,27,28]. Whereas tolerance appears to develop to the hyperthermic effects of TRPV1 antagonists, there does not appear to be tolerance to thermal hypoethetic effects. Collectively, these findings substantiate the role of TRPV1 as a logical mechanism associated with thermal hypersensitivity.

Although warm/hot thermal sensitivities represent some of the symptomatology, chronic pain is also characterized by allodynia, hyperalgesia and spontaneous pain where substantial heterogeneity in responsiveness to sensory stimuli exists. Using quantitative sensory testing stimuli, neuropathic patients can be sensitive to multiple stimuli including blunt pressure, pinprick, heat, cold, brushing of the skin, paradoxical heat sensation and enhanced pinprick wind-up, a form of central sensitization [29]. Moreover, gains of nociceptive sensitivity (hyperalgesia), loss of nociceptive sensitivity (hypoalgesia) or combinations of both are often observed, with some disease populations demonstrating differing phenotypes. For example, in a study by Maier *et al.* [29], 25% of peripheral nerve injury patients had hyperalgesia to hot pain, whereas 16% had hypoalgesia to this same stimulus. Until clinical trials for the effectiveness of TRPV1 antagonists are conducted, the generality of TRPV1 for pain conditions that are primarily non-inflammatory will remain unknown. Herein review and summarize the role of TRPV1 in stimulus modalities beyond warm thermal and whether sensitization to one sensory modality (e.g., thermal) could contribute to altered sensitivities to other modalities.

## 2. Expression Changes in TRPV1 Channels in Chronic Pain Conditions

Numerous preclinical studies suggest that TRPV1 expression is altered under conditions of chronic pain. For example, in normal animals, TRPV1 is predominately expressed in small sensory C-fibers and to a lesser extent in Aδ-fibers [4], both of which terminate in the spinal dorsal horn, where TRPV1 is localized to both pre- and post-synaptic neurons (and glial cells) in lamina I and II [5]. Following nerve injury, TRPV1 is down-regulated in the spinal cord after rhizotomy [4] and in the somata of damaged dorsal root ganglion (DRG) nerves two weeks following nerve transection or spinal nerve ligation (SNL) [30]. Despite this loss of TRPV1 in axotomized DRGs, TRPV1 was detected proximal to the neuronal site of lesion. After partial sciatic nerve ligation (PSNL), TRPV1 protein was increased in a population of undamaged DRG neurons [30]. Similarly, after lumbar (L) 5 SNL, TRPV1 expression was decreased in the damaged L5 DRG, whereas it was increased in the non-ligated L4 DRG, with a 3-fold increase expression observed in A-fibers. These findings were corroborated by independent laboratories [31,32,33]; however, see [34,35].

Changes in TRPV1 expression were also observed in the chronic constriction injury (CCI) model of neuropathic pain. TRPV1 expression increased by 149% and 167% in the ipsilateral spinal cord seven and 14 days, respectively, after injury, whereas no changes in expression were observed at earlier time points (one or three days) or in the contralateral spinal cord [36]. At day 14, capsaicin-evoked calcitonin gene-related peptide (CGRP) release was significantly higher (170%) in spinal cord slices from CCI animals compared to sham animals, suggestive that increased expression has functional effects on spinal sensitization. Thus, it has been hypothesized that increased TRPV1 expression, and its enhanced activity due to phosphorylation by local injury and glial derived inflammatory mediators, could contribute to spontaneous neuronal activity by reducing the thermal threshold, whereby TRPV1 becomes activated at body temperature [17,18].

With respect to osteoarthritis, which initially begins with a peripheral inflammatory component, preclinical studies suggest that chronic osteoarthritis produces central sensitization phenomena similar to that observed in neuropathic pain models [37,38,39]. Only a few studies have evaluated TRPV1 expression under osteoarthritic conditions. In patients with osteoarthritis, TRPV1 is expressed on synovium, as well as synovial fibroblasts suggesting both a neuronal and a non-neuronal role of TRPV1 in this condition [9,40]. In rats, TRPV1 is expressed in DRG neurons and knee joint synoviocytes [41,42]. Additionally, in the mono-iodoacetate (MIA) model of osteoarthritis, joint afferents in the DRG, as determined by Fast Blue staining, expressed a greater amount of TRPV1 (72%) compared to normal joint afferents (54%) [40]. 

Lastly, preclinical studies and the clinical presentation of pain associated with chronic bone cancer suggest similarities to neuropathic pain [43,44,45]. In humans, TRPV1 is up regulated in osteoclasts from osteoporotic patients [46]. In mice, TRPV1 is expressed on sensory fibers in mineralized bone and bone marrow, DRGs and in the spinal cord [47]. In an osteosarcoma model of bone cancer, the percentage of TRPV1 positive neurons was increased by 7% in the DRG neurons of bone cancer mice compared to control mice [48]. Overall, the studies highlighted above provide compelling evidence for a pivotal role for TRPV1 channels in a variety of pain conditions.

## 3. Behavioral Changes in Chronic Pain Conditions

Numerous approaches for the behavioral evaluation of TRPV1 in pathologic pain have been used, including knockout, knockdown and antagonist strategies. In knockout studies, mice lacking TRPV1 show a reduced sensitivity to heat and reduced thermal hypersensivity in response to incision or to the administration of inflamogens, such as mustard oil, carrageenan and CFA [49,50,51,52]. TRPV1 knockout mice show normal responses to other stimulus modalities (e.g., tactile and pressure stimuli) and develop similar magnitudes of hypersensitivity to both thermal and tactile stimuli after PSNL [49]. These findings were substantiated in another study wherein a similar magnitude of mechanical hypersensitivity (thermal sensitivity was not evaluated) was observed after L5/L6 SNL in both knockout and normal mice [53]. In general, these knockout studies indicate that TRPV1 is exclusively a thermal transducer. However, in a bone cancer pain model, TRPV1 knock-out mice displayed approximately 40–50% less spontaneous pain behaviors and palpation-induced flinching compared to wild-type mice [47]. 

In contrast to most knockout studies, knockdown approaches have demonstrated that TRPV1 modulates stimulus modalities other than thermal. Following intrathecal administration of a TRPV1 selective small interfering RNA (siRNA), CCI-induced cold hypersensitivity (cold plate) was significantly reduced by approximately 50% for up to five days [54]. This effect was transient, as cold hypersensitivity returned to control levels after seven days. TRPV1 knockdown was demonstrated by a lack of capsaicin-induced behaviors following rectal application of capsaicin. Similarly, twice daily intrathecal administration of TRPV1 antisense (but not mismatch) oligonucleotides significantly (~50%), reversed L5/L6 SNL-induced tactile hypersensivity, as measured by an electronic von Frey apparatus [34]. Behavioral studies were also conducted with RNA interference (RNAi) against TRPV1 and directly compared with TRPV1 knockout animals. Consistent with previous studies, TRPV1 knockout mice developed tactile hypersensitivity, whereas mice treated with intrathecal RNAi did not develop tactile hypersensitivity [53]. Since knockdown strategies have greater similarity to antagonist approaches compared to knockout strategies, these contrasting results between knockout and knockdown studies suggest functional differences between gene deletion and gene silencing approaches. 

The TRPV1 antagonists capsazepine and *N*-(4-*tert*-butylphenyl)-4-(3-chloropyridin-2-yl)tetrahydropyrazine-1(2*H*)-carboxamide (BCTC) have been widely studied in models of chronic pain. Capsazepine exhibits distinct species differences and is ineffective in rodent models of inflammatory and neuropathic pain. However, in guinea pigs, capsazepine reversed hypersensitivity in inflammatory models and produced an 80% reversal in PSNL-induced mechanical (paw pressure) hypersensitivity [55]. BCTC partially (~50%) attenuated PSNL-induced tactile (von Frey filaments) hypersensitivity in rats [56]. Similar results were obtained in another study wherein intrathecally administered BCTC produced a modest, yet significant reversal of CCI-induced tactile (von Frey filament) hypersensitivity at the 30 min time point [36]. Using an electronic von Frey apparatus, thioxo-BCTC partially reversed (~70%) L5/L6 SNL-induced tactile hypersensitivity [34]. Whereas recent studies with capsazepine and BCTC support a role for TRPV1 in modulating tactile hypersensitivity, it should be noted that these compounds are not TRPV1 selective and have activities at other targets (e.g., calcium channels, nicotinic and TRPM8 receptors) that could mediate these effects. 

Consistent with their effects in inflammatory pain models, TRPV1 antagonists also attenuate thermal hypersensitivity in neuropathic pain models. The TRPV1 selective antagonist A-425619 completely attenuated thermal hypersensitivity 10 days after SNL surgery in mice [35]. Against non-thermal stimuli, A-425619 produced a 36% reversal of both L5/L6 SNL- and CCI-induced tactile (von Frey filament) hypersensitivity following i.p. administration in rats [57]. However, in another study, A-425619 and a structurally dissimilar antagonist A-840257 did not produce any significant reversal of L5/L6 SNL-induced tactile hypersensitivity at doses that attenuated CFA-induced thermal hypersensivity [58]. Although the central nervous system (CNS) penetration of A-840257 was not disclosed, A-425619 exhibited low (~5%) CNS penetration [57], which might limit its effects under neuropathic pain conditions that are known to be mediated by spinal and supraspinal processes [review see 59]. Consistent with this notion, intrathecal (i.t.) administration of A-425619 produced a 33% reversal of CCI-induced tactile hypersensitivity [57].

To the extent that bone cancer pain has a neuropathic component, Ghilardi *et al.* [47] have demonstrated that daily administration of JNJ-17203212, a prototype TRPV1 antagonist, attenuated spontaneous and palpitation-induced flinching by approximately 50% compared to vehicle treated mice. Consistent with these findings, a single dose of ABT-102 produced an 18–19% reversal of ongoing pain-related behaviors, spontaneous ambulation and palpitation evoked pain-related behaviors [60] in a bone cancer pain model. The effectiveness of ABT-102 on these endpoints increased to approximately 43–45% reversal after daily administration for 12 days, which was not due to compound accumulation.

TRPV1 antagonists have been evaluated in the MIA-induced model of osteoarthritic pain. In this model, i.p. A-425619 produced a relatively small (24%) normalization of weight bearing [57]. These researchers also studied the importance of CNS exposure and the role of central TRPV1 actions in the MIA osteoarthritis model. Two compounds exhibiting similar *in vitro* TRPV1 potency and oral potency for reversing CFA-induced thermal hypersensitivity were evaluated after central and systemic administration [61]. Similar potencies were obtained for both compounds after i.t. administration in the MIA model, using weight bearing as the behavioral measure. However, the CNS penetrant molecule (A-784168) was more potent than the poorly CNS penetrant molecule (A-795614) after oral administration. Moreover, A-784168 reversed MIA-induced weight bearing differences by 85%, ~78% and 65% after oral, i.t., or intracerebroventricular routes of administration, respectively [61]. Consistent with these results, ABT-889425 dose dependently reversed MIA-induced impairment of grip strength with a dose of 300 μmol/kg producing a complete reversal [62]. These results highlight the potential importance of peripheral, spinal and supraspinal TRPV1 receptors in pain conditions. 

Since patients with chronic pain disorders require repeated administration of medications, preclinical studies have instigated the effectiveness of TRPV1 antagonist following chronic dosing. In one study, ABT-102 was evaluated on hind limb grip strength after acute and chronic administration in the MIA osteoarthritis model [60]. Animals received ABT-102 daily at either a low, non-effective (5%) or high, partially-effective (47%) dose after a single administration. Following 12 days of dosing, the low dose produced a 62% improvement, whereas the high dose produced a 98% improvement in grip strength. These results were confirmed with another TRPV1 antagonist, A-995662, a single high dose of which produced an 84% reversal in hind limb grip strength compared to a 67% reversal with the non-steroidal anti-inflammatory drug celecoxib [38]. Following 12 days of dosing with a partially effective (22%) dose of A-995662, there was a significant restoration of grip force (91%), which was not due to compound accumulation. Overall, chronic dosing appears to alter the effectiveness of TRPV1 antagonists, with changes in behavioral responses correlated with significant reductions in capsaicin-induced glutamate (26% decrease) and CGRP (41% decrease) release in the dissociated (rat) spinal cords. 

In addition to the contribution of TRPV1 to mechanical hypersensivity as measured through behavioral endpoints, other studies have provided evidence that mechanical hypersensitivity can be observed at the level of the nociceptive neuron. Increases in spontaneous firing and mechanically evoked firing of spinal wide dynamic range neurons have been observed in inflammatory pain models (*i.e.*, CFA), as well as MIA model of OA pain. The TRPV1 antagonist A-889425 attenuated both spontaneous and mechanically evoked firing in WDR and nociceptive specific neurons when evaluated at the level of the dorsal spinal cord after von Frey stimulation of the OA knee (62). Changes in neuronal activity also have been observed in other chronic inflammatory pain models likely containing a neuropathic component such as sickle cell disease [63,64]. Mice expressing the human sickle hemoglobin (HbSS) exhibit pathology and behavioral phenotypes consistent with human sickle cell patients. In mice, behavioral hypersensivity was observed with tactile, cold thermal and warm thermal stimuli. Along with attenuating these behavioral effects, the TRPV1 antagonist A-425619 substantially attenuated mechanical neuronal discharges in sensitized C-fibers to levels similar to control [64].

## 4. Allelic Variations in TRPV1 Channels

A growing body of research has underscored gender, psychological, cultural and genetic factors contributing to pain perception. For TRPV1, a number of allelic variants have been identified and the allele frequency can vary substantially among ethnic populations. For example, the TRPV1 1911A > G (rs8065080, I585V) minor allele in Caucasian populations is a major allele in Han Chinese and Japanese populations [65]. In female subjects with this variant, higher cold withdrawal tolerance to experimental cold water hand emersion has been reported [66] (however, see [67]). Moreover, this allele variant was associated with a significantly lower risk of painful knee osteoarthritis (OA) by comparing symptomatic and asymptomatic OA patients. However, because of its expression profile, it was also hypothesized to play a role in OA-induced cartilage morphology in addition to effects on nociception [68]. In neuropathic pain patients, this allele variant was associated with diminished cold sensitivity and in a subset of patients characterized by preserved sensory function, significantly less heat hyperalgesia, pinprick hyperalgesia and mechanical hypaesthesia [69]. Importantly, while the genetic variant contributed to the diminished somatosensory hypersensitivity in neuropathic pain patients, it was not linked to the susceptibility for developing neuropathic pain. A similar finding was observed in that TRPV1 was not associated with susceptibility for developing chronic pancreatitis, though the impact on pain perception was not fully quantified [70]. Another TRPV1 allele variant 1103C > G (rs222747, M315I) was associated with cold hypaesthesia [69] suggesting that there might be additional TRPV1 genetic variability.

Whereas these genetic association studies suggest a role for TRPV1 variants in multiple clinical pain states, *in vitro* studies have not demonstrated robust differences in channel function among alleles. For example, the TRPV1 1911A > G allele had normal response to capsaicin, pH and temperature [71] and other alleles evaluated *in vitro* by Xu *et al.* [65] had similar EC_50_ values for capsaicin compared to TRPV1 wild-type control. However, slight variations were observed with some alleles including changes in the Hill slope coefficient and maximal responsiveness to anandamide. The later effect appeared related to increased TRPV1 expression in cells caused by the allele *in vitro*. There is increasing recognition that gene expression caused by allelic variants, in the absence of functional changes, might contribute to human genetic variation [65]. In what is akin to a human knockout study, an individual was identified with complete insensitivity to capsaicin. Upon further evaluation, this individual had TRPV1 expression in the buccal mucosa that was only 38% of normal levels [72]. While this subject displayed capsaicin insensitivity, sensitivities to pH and water temperatures between 45 and 60 ºC were similar to normal subjects. Together with changes in distribution after neuronal injury, such results suggest that the level of TRPV1 expression has profound consequences on neuronal sensitivities.

## 5. Conclusions

The TRPV1 channel is thought to be a primary mediator of warm thermal sensation. Recent studies indicate that inflammatory and neuronal injury alters the localization of TRPV1 and sensitizes the channel to respond to stimulus modalities beyond the classical thermal profile. Preclinical localization and expression studies provide evidence that changes in TRPV1 occurs at multiple levels (e.g., peripheral nociceptors, DRG and dorsal spinal cord) along pain pathways after neuronal injury. In studies that have evaluated functional activity, a gain of TRPV1-mediated currents is typically observed, which is related to local changes in pro-inflammatory neurotransmitters, cytokines and secondary messengers responsible for sensitization of the TRPV1 channel. In relation to allelic variants, such changes suggest that the TRPV1 channel may play a role in the variability associated with pain sensation, perception and the development of hypersensitivities under disease conditions. This heterogeneity among patient populations may be important for the continued development of novel analgesics. While functional differences among alleles have not demonstrated robust changes in sensitivity to activators of TRPV1, published studies have not evaluated whether the TRPV1 antagonists under clinical development have differing profiles in patient populations. Potential differences in sensitivities to antagonists, including the observation of thermal hypoethsia and hyperthermia might be observed. These initial insights suggest a complex role of TRPV1 in somatosensory pain perception and emphasize the need for a greater understanding of such processes with an eye towards identifying approaches for stratifying patients for better, more personalized, evidence based therapies.

## Appendix

**Table 1:** Summary of TRPV1 Expression in Pain Models.

Reference	Species	Model	Changes in Expression
**[10]**	human	IBD	In rectosigmoid biopsies from IBD patients with pain, TRPV1 expression was significantly (4 to 5-fold) higher compared with controls and IBD patients without pain. In IBD patients with pain, TRPV1 expression correlated with abdominal pain severity.
**[7]**	mouse	CFA	CFA inflammation increased TRPV1 expression 3-fold in IB4-positive DRG neurons. In these neurons, capsaicin-sensitivity increased 3-fold and proton-sensitivity increased nearly 2-fold.
**[41]**	mouse	normal	TRPV1 is localized within periosteum of periarticular bone, the articular capsule and vasculature of joints. TRPV1 expression occurs in 43% and 39% of DRG neurons innervating joint afferents the ankle and knee, respectively.
**[34]**	rat	SNL	TRPV1 expression decreased in ligated L5 and L6 but not in the non-ligated L4 DRG and dorsal spinal cord.
**[5]**	rat mouse	normal	TRPV1 expression is seen in lamina I and II of the L4 dorsal spinal horn and in spinal glial cells but not in TRPV1 KO mice.
**[9]**	human	OA/RA	TRPV1 is expressed in synovial fibroblasts from OA and RA patients (no comparison with non-OA/RA patients).
**[40]**	rat human	MIAOA	Following MIA, TRPV1 expressing DRG neurons is increased (72%) compared to control (54%); occasional TRPV1 positive fibers are observed in rat and human knee joints
**[31]**	rat	SNL	TRPV1 immunoreactivity and mRNA was higher in the L4 DRG ipsilateral (~37–45%) compared to contralateral (~25–32%) 1 d to 28 d after SNL. Expression correlated with heat hypersensitivity.
**[47]**	mouse	BCP	TRPV1 expression was localized on sensory femur DRG neurons. No expression changes were observed in neurons of sarcoma-bearing femurs.
**[16]**	human	chronic cough	TRPV1 expression of bronchial epithelial nerves in patients with chronic cough was 4-fold higher than controls. Patients with chronic cough were 30-fold more sensitive to the tussive effects of capsaicin.
**[12]**	human	esophageal reflux	TRPV1 mRNA and protein is increased 2- to 3-fold in the esophageal mucosa of patients with non-erosive reflux disease and erosive esophagitis compared to controls.
**[4]**	rat	rhizSNL	TRPV1 expression was observed in DRG of small to medium diameter neurons and lamina I/II. After rhiz, expression decreased distal and increased proximal to SNL lesion.
**[73]**	rat	STZ	DRGs from STZ neurons demonstrated decreased homogenate TRPV1 immunoreactivity (−10%), increased plasma membrane TRPV1 (+151%), TRPV1 phosphorylation (+256%), capsaicin-induced currents (+45%) and pH-evoked currents (+43%) 4–8 weeks after STZ.
**[30]**	rat	AXO, PSNL, SNL	TRPV1 expression 2 weeks after AXO, PSNL, and SNL was decreased in DRGs of damaged nerves (except proximal to lesion), increased in DRG of undamaged neurons and increased 3-fold in A-fibers.
**[36]**	rat	CCI	TRPV1 expression increased by 149% and 167% in the ipsilateral spinal cord 7 d and 14 d, respectively, after CCI injury. No changes in expression were observed at earlier time points (1 d or 3 d) or in the contralateral spinal cord (14 d). Capsaicin-evoked CGRP release was significantly higher (170%) in spinal cord slices from CCI animals compared to sham animals.
**[15]**	human	chronic cough	TRPV1 expression is higher in airways from patients with chronic cough compared to controls.
**[32]**	rat	SNL	Percentage of CB1 neurons co-labeled with TRPV1 increased from 25% to 59% 2 weeks after SNL
**[48]**	mouse	BCP	TRPV1 mRNA and protein increased 190% and 290%, respectively, in the DRGs of sarcoma-treated mice compared to sham controls.
**[51]**	mouse	STZ	In dissociated DRG neurons from STZ-treated mice, peak capsaicin-induced currents where significantly higher (1.9-fold) in thermally hypersensitive mice and significantly lower (0.6-fold) in thermally hyposensitive mice compared to age-matched controls. [3H]-RTX binding in DRG and paw skin was significantly increased (2.6 and 1.8-fold, respectively) in thermally hypersensitive mice and significantly lower (0.3 and 0.5-fold, respectively) in thermally hyposensitive mice compared to age-matched controls.
**[74]**	mouse	STZ	STZ has direct action on neurons, which up-regulates TRPV1 expression and increases capsaicin-induced currents
**[14]**	human	CPP	TRPV1 expression was 7-fold higher in pelvic tissues from patients with CPP compared to controls, which was not due to an increase in neuronal fibers.
**[33]**	mouse	PSNL	In neonatal capsaicin-treated mice, TRPV1 expression is absent but increases in A-fiber DRG neurons after PSNL
**[46]**	human	OA	TRPV1 mRNA is increased 5-fold in osteoclasts from osteoporotic and osteoporotic women compared to normal menopausal women. Capsaicin was less potent and produced a decreased Ca^2+^ response in osteoclasts from osteoporotic and osteoporotic women compared to normal menopausal women.
**[42]**	rat	MIA	TRPV1 is expressed in DRG neurons and knee joint synovium of control and MIA-treated rats. No quantitative differences between groups evaluated.
**[13]**	human	GERD	TRPV1 mRNA and protein were significantly increased in patients with erosive esophagitis compared to asymptomatic and healthy control patients.
**[35]**	mouse	SNL	Injury increased the percentage of heat sensitive small-diameter IB4 positive isolated DRG neurons (13% control *vs.* 56% SNL) and conversely decreased the percentage of heat sensitive small-diameter IB4 negative isolated DRG neurons (66% control *vs.* 34% SNL). A-425619 blocked heat sensitivity in both subsets of DRG neurons.
**[75]**	rat	CCI	CCI increases TRPV1 protein but not mRNA in DRGs 7 d after surgery.
**[11]**	human	IBD	TRPV1 expression was 2-fold higher in patients with IBD compared to healthy controls.

AXO, axonomy; BCP, bone cancer pain; Carr, carrageenan; CCI, chronic constriction injury; CFA, complete Freund’s adjuvant; CPP, chronic pelvic pain; DRG, dorsal root ganglion; GERD, gastro-esophageal reflux disease; IBD, irritable bowel disorder; MIA, mono iodoacetate; OA, osteoarthritis; PSNL, partial sciatic nerve ligation; RA, rheumatoid arthritis; Rhiz, rhizotomy; SNL, spinal nerve ligation; STZ, streptozotocin.

**Table 2:** Summary of TRPV1 Antagonists in Chronic Pain Models.

Reference	Model	Species	Compound (Dose; Route; Frequency; Duration)	Stimulus and Effects
**[54]**	CCI	rat	siRNA (1 μg; i.th.)	Decreased CCI-cold (acetone) hypersensitivity by 50% and blocked capsaicin-induced behaviors.
**[34]**	SNL	rat	AS ODN (45 μg *b.i.d.*; i.th.)	Partially reversed SNL tactile (e-von Frey) hypersensitivity.
**[34]**	SNL	rat	thioxo-BCTC (2.15–21.5 mg/kg; i.v.)	Reduced tactile (e-vonFrey) hypersensitivity by ~70% at high dose (ED_50_ value of 10.6 mg/kg).
**[53]**	SNL	mouse	TRPV1 shRNAtg	TRPV1 shRNAtg mice did not develop tactile (e-von Frey or von Frey) hypersensitivity and had significantly decreased latencies on the 48 ºC and 58 ºC hotplate.
**[63]**	MIA	Rat	A-889425 (10–300 μmol/kg; p.o.); (10–30 μmol/kg; i.v.)	A-889425 completely reversed MIA-induced impaired grip strength and attenuated evoked and spontaneous firing by 44% and 61%, respectively, compared to baseline.
**[61]**	MIA	rat	A-784168 (3–30 μmol/kg; p.o.); (10–100 nmol; i.th.) A-795614 (30–300 μmol/kg; p.o.); (10–100 nmol; i.th.)	A-784168 significantly reversed MIA-induced weight bearing differences with an ED_50_ value of 8 μmol/kg, p.o and 22 nmol, i.th. A-795614 significantly reversed MIA-induced weight bearing differences with an ED_50_ value of 280 μmol/kg, p.o. and 26 nmol, i.th.
**[47]**	BCP	mouse	JNJ-17203212 (30 mg/kg; s.c.; *b.i.d* for 8–12 d)	TRPV1 KO and JNJ-17203212 attenuated spontaneous (~50%) and palpation-induced (~50%) flinching. JNJ-17203212 attenuated palpitation-induced increases in spinal lamina I-II c-Fos expression (7.5 cFos-IR) compared to vehicle (17.5 cFos-IR).
**[64]**	SCD	mouse	A-425619 (100 μM/kg; i.p.)	A-425619 significantly attenuated tactile (von Frey) hypersensitivity (30–90 min) in mice expressing human sickle hemoglobin in erythroid cells compared to vehicle treated mice. Electrophysiology *ex vivo* skin preparations from mice had C- and high threshold Aδ-fiber sensitization to mechanical force that was attenuated by A-425619.
**[57]**	MIA CCI SNL	ratmouse	A-425619 (30–300 μmol/kg; i.p.)	A-425619 reversed MIA weight-bearing to 47% of normal weight distribution and reversed von Frey tactile hypersensitive 2 weeks after SNL (36% reversal) or CCI (36% reversal) surgery.
**[60]**	MIABCP	ratmouse	ABT-102 (3–100 μmol/kg, p.o.; single dose or *b.i.d.* for 12 d) A-993610 (μmol/kg, p.o. single dose or *b.i.d.* for 12 d)	Acutely, ABT-102 significantly reversed MIA-induced difference in weight bearing (ED_50_ = 30 μmol/kg) and grip strength (ED_50_ = 10 μmol/kg). ABT-102 significantly reversed CBP-induced spontaneous guarding by 85%, decreased ambulation by 85% and palpation induced pain by 65%. Doses of ABT-102 that had minimal effects acutely were more effective following chronic administration and significantly reversed MIA-induced decreased grip strength, bone cancer decreased spontaneous ambulation, ongoing pain and palpation evoked pain. Similar improvements in pain behaviors were observed after acute and chronic A-993610. Effects were not due to drug accumulation.
**[58]**	SNL	rat	A-425619 (3–30 mg/kg; i.p.) A-840257 (3–30 mg/kg; i.p.)	A-425619 and A-840257 lacked effects on von Frey tactile hypersensitivity in the SNL model of neuropathic pain.
**[36]**	CCI	rat	BCTC (30–300 nmol; p.o.)	100 and 300 nmol BCTC produced a modest, though significant reversal of CCI-induced tactile (von Frey) hypersensitivity 30 min (but not 60 or 120 min) after administration.
**[62]**	CFA		A-889425 (10–100 μmol; i.p.)	A-889425 significantly attenuated CFA-induced tactile hypersensitivity at 30 and 100 μmol. Electrophysiology recordings from WDR neurons had significantly greater spontaneous and evoked firing, which was attenuated by A-889425 administration.
**[48]**	BCP	mouse	I-RTX (0.03–1 μmol; i.p.)	I-RTX significantly decreased spontaneous flinching, attenuated decreased ambulation and reversed weight-bearing differences during ambulation in sarcoma-treated mice.
**[56]**	PSNL	rat	BCTC (1–30 mg/kg; p.o.)	BCTC partial attenuated PSNL von Frey tactile hypersensitivity (~50%) with significant reversal at 10 and 30 mg/kg.
**[38]**	MIA	rat	A-995662 (3–100 μmol/kg; p.o.; single dose or b.i.d. for 12 d)	Acute doses of A-995662 significantly reversed MIA-induced decreased grip strength. An acutely sub-effective (22% reversal) dose significantly restored grip force (91% reversal) after chronic administration. The duration of effectiveness was longer than the detection of compound in brain or plasma.
**[35]**	SNL	mouse	A-425619 (200 μmol/kg; i.p.)	A-425619 completely attenuated SNL-induced radiant heat thermal hypersensitivity.
**[55]**	CFA, PSNL	guinea pig, rat, mouse	capsazepine (10–100 mg/kg; s.c.)	Rodent species were insensitive to capsazepine reversal of hypersensitivity in inflammatory and neuropathic models. In guinea pigs, capsazepine produced an 80% reversal of PSNL-induced mechanical hypersensitivity.

AS ODN, antisense oligonucleotides; BCP, bone cancer pain; CFA, Complete Freund’s Adjuvant; CCI, chronic constriction injury; b.i.d.; twice daily; d, day; e-von Frey, electronic von Frey; IR, immunoreactivity; i.p., intraperitoneal; i.th., intrathecal; KO, knock-out; MIA, mono iodoacetate; PSNL, partial sciatic nerve ligation; p.o., per os; SCD, sickle cell disease; siRNA, small interfering RNA; shRNAtg, small hairpin RNA transgenic; SNL, spinal nerve ligation; s.c., subcutaneous; WDR, wide dynamic range.
